# Global pharmacovigilance reporting of the first monoclonal antibody for canine osteoarthritis: a case study with bedinvetmab (Librela™)

**DOI:** 10.3389/fvets.2025.1558222

**Published:** 2025-04-24

**Authors:** Beatriz P. Monteiro, Anthony Simon, Oliver Knesl, Kristen Mandello, Steven Nederveld, Natasha J. Olby, John F. Innes, B. Duncan X. Lascelles

**Affiliations:** ^1^Global Medical Affairs, Zoetis, Parsippany, NJ, United States; ^2^Veterinary Medicine Research and Development, Global Pharmacovigilance, Zoetis, Parsippany, NJ, United States; ^3^Department of Clinical Sciences, College of Veterinary Medicine, North Carolina State University, Raleigh, NC, United States; ^4^Comparative Medicine Institute, North Carolina State University, Raleigh, NC, United States; ^5^Movement Referrals Independent Veterinary Specialists, Halton, United Kingdom; ^6^Comparative Pain and Orthopedic Research Laboratories, College of Veterinary Medicine, North Carolina State University, Raleigh, NC, United States

**Keywords:** bedinvetmab, canine, chronic pain, dogs, nerve growth factor, osteoarthritis, pharmacovigilance, safety

## Abstract

**Introduction:**

Continuous product monitoring post approval builds on the knowledge gained during clinical studies to aid in understanding a product’s safety and efficacy profile. Pharmacovigilance reporting of a medicinal product might be influenced by several factors including duration in the market, geographical region and veterinary practices. The goals of this report are to present the global data accrued for bedinvetmab, the first monoclonal antibody for canine osteoarthritis, and to explore reporting patterns globally and across major markets.

**Methods:**

Adverse event reports from the Zoetis Global Pharmacovigilance database (from first introduction on 01 February 2021 through 30 June 2024) were collected irrespective of suspected causality or off-label use. Each adverse event was coded using the Veterinary Dictionary for Drug Related Affairs (VeDDRA) terminology. The top 20 most reported VeDDRA terms were identified. Countries were ranked by number of doses distributed and frequency of adverse events.

**Results:**

Globally, 18,102,535 doses of bedinvetmab were sold during the study period with a total of 17,162 adverse events reported in dogs (9.48 events/10,000 treated animals (doses)). Eight clinical signs were considered rare (1–10 events/10,000 treated animals (doses)) with lack of efficacy having the highest rate (1.70) followed by polydipsia, ataxia, polyuria/pollakiuria, anorexia, lethargy, death, and emesis. All other clinical signs were considered very rare (< 1 event/10,000 treated animals (doses)). Median (interquartile range) of dogs’ age and body weight were 12 (10–13) years and 26 (16–34.6) kg, respectively. The top eight countries by market size were United States (US), United Kingdom (UK), Germany, Spain, France, Italy, Canada, and Australia; from these, the top five by frequency of adverse events were Canada, US, UK, Australia and Germany. The most reported adverse events following bedinvetmab are considered rare or very rare.

**Discussion:**

The reported clinical signs generally aligned with expected adverse events or were anticipated within the population receiving bedinvetmab. Reporting rates and patterns in general and for specific VeDDRA terms greatly varied between countries and were not related to market size. Most dogs for which adverse events were reported were considered older and in fair clinical condition. Reporting to pharmacovigilance contributes to the understanding of the safety profile of a medicinal product.

## Introduction

Osteoarthritis (OA) is a highly prevalent disease affecting dogs of all ages, sizes and breeds (1, 2). It is a chronic, degenerative condition of synovial joints characterized by low-grade inflammation (3) and progressive deterioration of all joint tissues. Osteoarthritis is clinically important because it can be associated with pain and mobility impairment, both of which can manifest from mild to severe and fluctuate over time. Pain associated with OA limits dogs’ ability to enjoy previously rewarding activities (e.g., going for walks, playing with other animals, greeting their caregivers at the door) negatively impacting their quality of life (QoL) and the bond with their caregivers.

Treatments for pain associated with OA primarily aim to manage pain and improve QoL, with non-steroidal anti-inflammatory drugs (NSAIDs) being the only category of therapeutics associated with robust evidence of efficacy until recently. However, NSAIDs can have limited efficacy (4) and can cause significant adverse events (5, 6), necessitating the search for alternative treatments. Bedinvetmab (Librela™, Zoetis) is a new therapeutic approach that represents a significant development in OA pain management as a monthly subcutaneous treatment. It is a fully canine monoclonal antibody (mAb) that sequesters nerve growth factor (NGF), a protein which plays a key role in OA pain (7–10), thereby preventing NGF’s pro-nociceptive effects. Nerve growth factor is elevated in osteoarthritic joints (11) and binds to the receptors tropomyosin receptor kinase A and p75 neurotrophin, leading to processes that cause sensory nerve sensitization, contribute to local inflammation and result in hyperinnervation (12). By binding to NGF, bedinvetmab prevents its interaction with the receptors, thereby disrupting the pain signaling pathway and reducing the hyperalgesic state associated with OA (10). Bedinvetmab was first authorized for commercialization by the European Commission following a positive opinion from the European Medicines Agency (EMA) and licensed in the European Union on 10 November 2020.

Both human and animal health products introduced by an appropriate sponsor (e.g., pharmaceutical company) undergo stringent approval processes by regulatory agencies such as EMA, Food and Drug Administration (FDA), and Veterinary Medicines Directorate (VMD), although there are key differences. For human health products, the process includes pre-clinical studies in animals and multiple phases of clinical trials in humans. For animal health products, the process generally focuses on testing in the target species and may or may not include studies in laboratory animals. All data generated in the development process are submitted to the regulatory agency as part of a dossier for review by a team of professionals with various expertise including veterinarians, animal scientists, biostatisticians, chemists, microbiologists, pharmacologists, and toxicologists. Upon thorough review, if the regulatory agency deems the submitted dossier demonstrates adequate quality, safety and efficacy, the drug is approved, and the sponsor is legally allowed to market and sell the product in that territory.

The relatively small size and limited diversity of the dog population treated in pre-approval safety and efficacy studies means that only the more frequently occurring adverse events will likely be identified. Thus, a product’s overall safety profile is inevitably composed of both high-quality comparative pre-approval studies and uncontrolled (often of lower quality or limited data), post approval spontaneously reported adverse event data. An adverse event is defined by the International Cooperation on Harmonization of Technical Requirements for Registration of Veterinary Medicinal Products (VICH) (13) as ‘*any observation in animals, whether or not considered to be product-related, that is unfavourable and unintended and that occurs after any use of veterinary medicinal product (VMP) (off-label and on-label uses)*’. Included are events related to a suspected lack of expected efficacy according to approved labeling or noxious reactions in humans after being exposed to VMPs. A valid adverse event is a case for which the minimum of a reporter, product, patient and problem can be identified. This includes cases which at a later date may be found or assessed as unrelated to product. An *adverse event* may be concluded by a Regulatory Authority to be an *adverse reaction* when there is at least a reasonable possibility (i.e., relationship cannot be ruled out) that harmful and unintended observations were a response to the VMP administered at doses normally used in animals for prophylaxis, diagnosis or therapy of disease or for modification of physiological function (13). This implies that there is a requirement to evaluate adverse events reported in aggregate, considering the source, data quality, and validity to determine if there is at least a reasonable possibility of causal associations. Importantly, the accuracy of information is dependent on the quality of information provided by the individual reporting the event. Adverse events may also be related to underlying or pre-existing diseases, concomitant product use, or other related causes; thus, for any individual adverse event report, there is no certainty that the reported product caused the adverse event.

Pharmacovigilance is defined as the science and activities relating to the detection, assessment, mitigation, and prevention of adverse events after a drug is introduced onto the market. These activities build on the knowledge gained during the clinical studies to aid in understanding a product’s safety and efficacy profile after expanded distribution, when many more patients are exposed and rare potentially product related risks can be detected and evaluated. Through continuous monitoring of both individual adverse events and aggregate case listings over time, trends related to patient population, environmental or market factors may become evident and provide additional insights to protect animal health and welfare and the safety of people exposed to the products (13–15). Seasonality of a disease process or natural progression of a disease for which a therapy is a palliative treatment rather than a cure may also demonstrate repeatable periods of higher rates of reporting or an increase in perceived lack of efficacy over time, respectively.

Different countries have different structures and processes for veterinary pharmacovigilance, but all processes aim to collect and analyze adverse events, and rely on the collaboration of regulatory agencies, pharmaceutical companies, veterinarians, and animal caregivers. The commercial party responsible for marketing a VMP and for collecting, storing, and reporting of adverse events to regulatory agencies is known as a Marketing Authorization Holder (MAH). Different agencies oversee veterinary pharmacovigilance by region or country. For example, the FDA in the United States (US), the EMA in the EU, the VMD in the United Kingdom (UK), the Australian Pesticides and Veterinary Medicines Authority in Australia or the Ministry of Agriculture, Forestry, and Fisheries in Japan. In most countries the usual route of reporting is directly from the veterinarian or animal caregiver to the MAH but in many European countries a substantial proportion of reports may be sent directly to the local country regulatory agency. Although most countries and regions have similar guidelines for regulating VMPs, there can be noticeable differences in the adverse event reporting cultures of veterinarians or animal caregivers. Challenges in reporting adverse events include underreporting (16), varying regulatory requirements, and differences in veterinary practice. Regional differences in the prevalence of certain diseases, animal husbandry practices, the use of VMPs, social and cultural differences, and more recently, social media, can all influence adverse event reporting (17). There may be delays between the approval of a product by a regulatory agency and the first reports of adverse events to the MAH or a regulatory authority. Product launch can take weeks to months after approval. These factors can delay the timing of any adverse event report.

The goals of this study are to present the data accrued in the Zoetis Global Pharmacovigilance database (ZGPVDB) from launch of bedinvetmab in Europe on 01 February 2021 to 30 June 2024 and to explore reporting patterns globally and across major markets for bedinvetmab, the first mAb for canine OA pain and one of the first three in veterinary medicine. Specifically, the research questions were “What are the most common adverse events reported for bedinvetmab in dogs?” and “Are there differences in pharmacovigilance reporting across different countries or regions?”

## Materials and methods

### Data source

Data were collected using commercially available software (PV Works by ENNOV) and added to the ZGPVDB which is the central validated pharmacovigilance database for all of Zoetis’ products sold throughout the world. The minimum dataset for a valid reportable adverse event is a person (i.e., reporter), a product, a patient, and a problem, known as the four ‘Ps’. The MAHs try to collect as full a data set as possible for each adverse event, but this is often limited by the information provided, knowledge / information available to the reporter (in the case of animal caregivers) or lack of follow up information. Adverse event reports were collected, verified, evaluated, and reported as part of the company’s regulatory obligations. Unsolicited adverse events for bedinvetmab were submitted by various stakeholders including veterinarians, veterinary staff, animal caregivers, non-Zoetis social media, regulatory authorities, and through surveillance of published literature. Active monitoring of Zoetis’ social media accounts was done as this is a regulatory requirement. When any reference to an adverse event was available in Zoetis owned social media accounts, the reporter was contacted, and additional information was requested to create a report. In addition, the EMA’s EudraVigilance Veterinary (EVVet) database is regularly reviewed and assessed by Zoetis Global Pharmacovigilance to identify cases involving bedinvetmab which have been submitted by European National Competent Authorities (NCAs) or by other MAHs as part of a report involving one of their own products. Efforts are made to identify and manage any duplicate cases to avoid double counting of data; however, duplicate identification is confounded by data privacy rules as no contact information is available for any imported case. Close communication is maintained between the global Zoetis teams responsible for entering adverse event reports in individual countries, Global Pharmacovigilance, Manufacturing, and Regulatory Affairs, and numerous regulatory agencies to identify any emerging safety, efficacy or quality concern that may arise with a product, so they can be addressed appropriately.

### Data management and analysis

All reports received, irrespective of suspected causality or off-label use, from first launch on 01 February 2021, until 30 June 2024, were logged into the ZGPVDB and included in data analysis. Each adverse event was coded in a standardized format using Veterinary Dictionary for Drug Related Affairs (VeDDRA) terminology (18, 19) in English language. The VeDDRA system is organized as a 4-level hierarchical structure, with the highest-level being system organ class, followed by high level term, preferred term, and low-level term at the lowest level (18, 19). For each report, a dog could have one or several VeDDRA terms included. These reports were then submitted to regulatory agencies worldwide in accordance with the respective agencies’ requirements and Zoetis’ internal standard operating procedures. Adverse events, reported events, clinical signs or VeDDRA terms are used interchangeably across the manuscript.

The top 20 most reported adverse events (VeDDRA terms) were identified and the signalment of dogs from such reports were evaluated with regards to age, body weight and clinical condition (categorized as ‘unknown’, good’, ‘fair’, ‘poor’ or ‘critical’). The VICH guidance for the latter classifications is related to the attending veterinarian’s assessment of the dog’s health status prior to administration of the VMP. Adverse events were sorted by country or region and listed from highest to lowest rates. Countries were ranked by market size (number of doses distributed of bedinvetmab until 30 June 2024) and further exploration into the reported clinical signs (VeDDRA terms) of the top eight countries was performed. Countries were also ranked according to frequency of reporting of adverse events. Descriptive data were calculated using Microsoft Excel and data visualization were done with Tableau visual analytics platform (PV Works database).

Due to recent anecdotal reports of an unclassified arthropathy in a small number of dogs treated with bedinvetmab, the ZGPVDB was specifically searched to investigate musculoskeletal adverse events. The following keywords were searched: ‘RPOA (Rapidly Progressive OA)’, ‘rapid’, ‘progress’, ‘fracture’, ‘radiograph’, or the VeDDRA terms ‘arthritis’, ‘bone and joint disorder NOS (Not Otherwise Specified)’ or ‘joint pain NOS’.

## Results

### Adverse events reported globally

**Figure 1:**
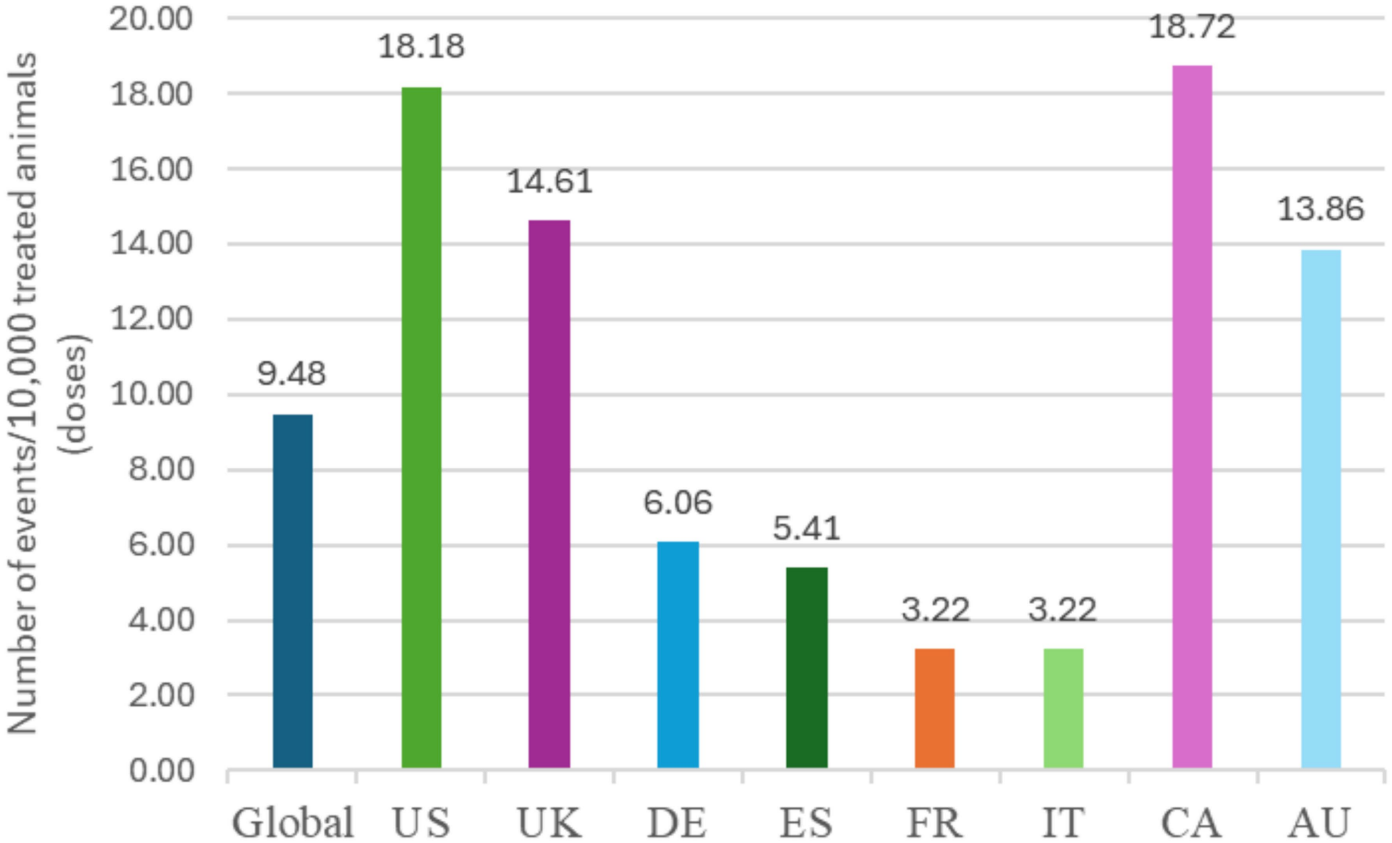
Frequency of overall reported events [Veterinary Dictionary for Drug Related Affairs (VeDDRA) terms] globally and for the top eight countries by market size from 01 February 2021 to 30 June 2024. CA, Canada; US, United States; UK, United Kingdom; DE, Germany; ES, Spain; FR, France; IT, Italy; CA, Canada; AU, Australia.

**Figure 2:**
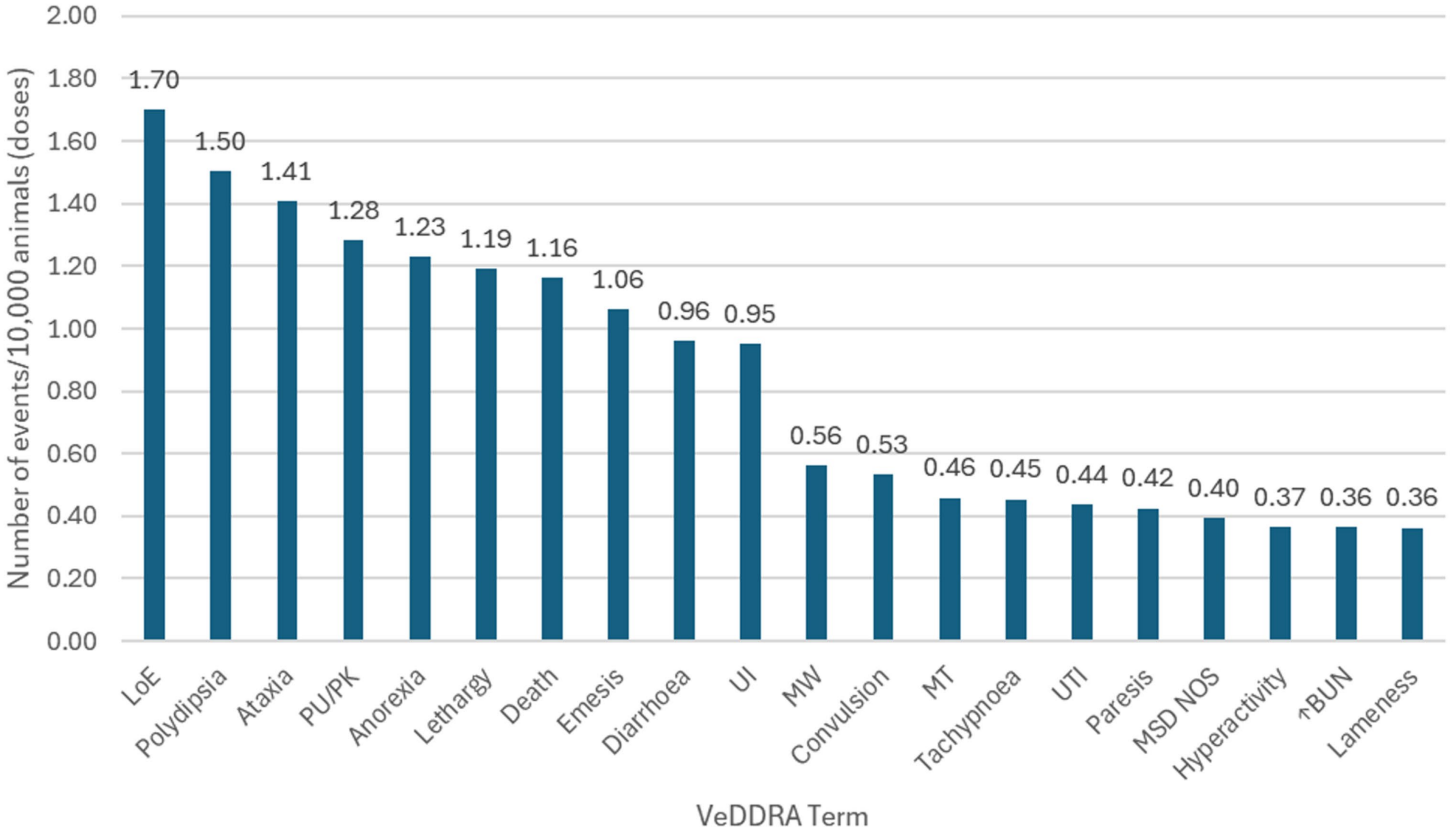
Frequency of the top 20 reported events [Veterinary Dictionary for Drug Related Affairs (VeDDRA) terms] globally for bedinvetmab from February 01st, 2021 to June 30th, 2024 and their classification as ‘rare’ or ‘very rare’. See Table 1 for description of categories of frequency of adverse drug reaction. LoE, Lack of Efficacy; PU/PK, Polyuria/Pollakiuria; UI, Urinary Incontinence; MW, Muscle Weakness; MT, Muscle tremor; UTI, Urinary Tract Infection; MSD NOS, Musculoskeletal Disorder Not Otherwise Specified; ↑BUN, Increased Blood Urea Nitrogen.

Globally, 18,102,535 doses of bedinvetmab were sold during the study period with a total of 17,162 adverse events reported involving 17,775 dogs (i.e., some events involved more than one dog) for an overall rate of 9.48 events/10,000 doses, including lack of efficacy reports (Figure 1). The most frequently reported signs were considered rare [between 1 and 10 events per 10,000 treated animals (doses)], with lack of efficacy having the highest rate at 1.70, followed by polydipsia, ataxia, polyuria/pollakiuria, anorexia, lethargy, death, and emesis (based on the number of doses sold; frequency calculations assume one treated dog per one dose sold) according to the guidance provided by the Council for International Organizations of Medical Sciences (CIOMS) (20, 21) (Figure 2, Table 1). All other reported clinical signs were considered very rare [< 1 event per 10,000 treated animals (doses)].

**Table 1:** Categories of frequency of adverse events according to the Council for International Organizations of Medical Sciences (CIOMS) (22).

Category	Frequency*	Percentage
Very common	≥ 1 event / 10 treated animals (doses)	≥ 10%
Common	> 1 to 10 events / 100 treated animals (doses)	≥ 1% and < 10%
Uncommon	1 to 10 events / 1,000 treated animals (doses)	≥ 0.1% and < 1%
Rare	1 to 10 events / 10,000 treated animals (doses)	0.01% and < 0.1%
Very rare	< 1 event / 10,000 treated animals (doses)	< 0.01%

*Frequency calculations assume one treated dog per one dose sold.

**Figure 3:**
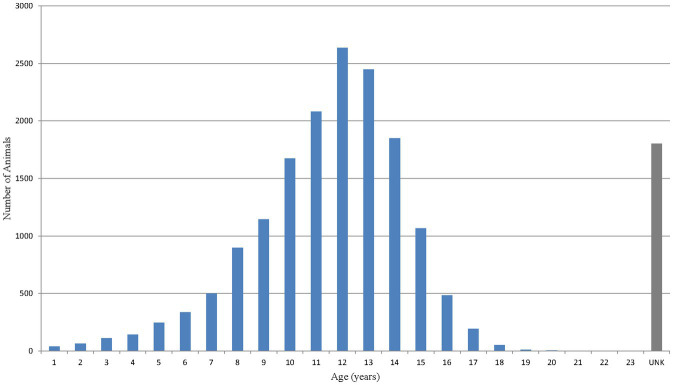
Age distribution of dogs based on number of reported events [Veterinary Dictionary for Drug Related Affairs (VeDDRA) terms] for bedinvetmab globally from February 01st, 2021 to June 30th, 2024.

Age was not recorded for approximately 10% of dogs (1,803 out of 17,775). Excluding these, the mean age of dogs with a reported adverse event was 11.4 years with a median (interquartile range) of 12.0 (10.0–13.0) years (Figure 3). Nearly 80% of dogs (13,805 out of 17,775) experiencing an adverse event were either ≥10 years of age or listed as ‘unknown age’. Dogs were predominantly listed to be in ‘fair’ condition prior to treatment with bedinvetmab. The ‘fair’ condition was related to the presence of OA, co-morbidities or any combination of conditions often reported in older dogs. Body weight was not recorded for approximately 16% of dogs (2,799 out of 17,775). Excluding these, the median (interquartile range) of dogs’ weight was 26.06 (16.0–34.6) kg (data not shown). For comparison, the mean age, body weight and clinical condition from the entire canine ZGPVDB are 4.7 years, 18.4 kg, and ‘good’, respectively.

When looking specifically at musculoskeletal adverse events or reported events that included radiographs, a total of 2,404 cases were identified [1.33 events/10,000 treated animals (doses)].

### Regional differences in adverse event reporting

**Figure 4:**
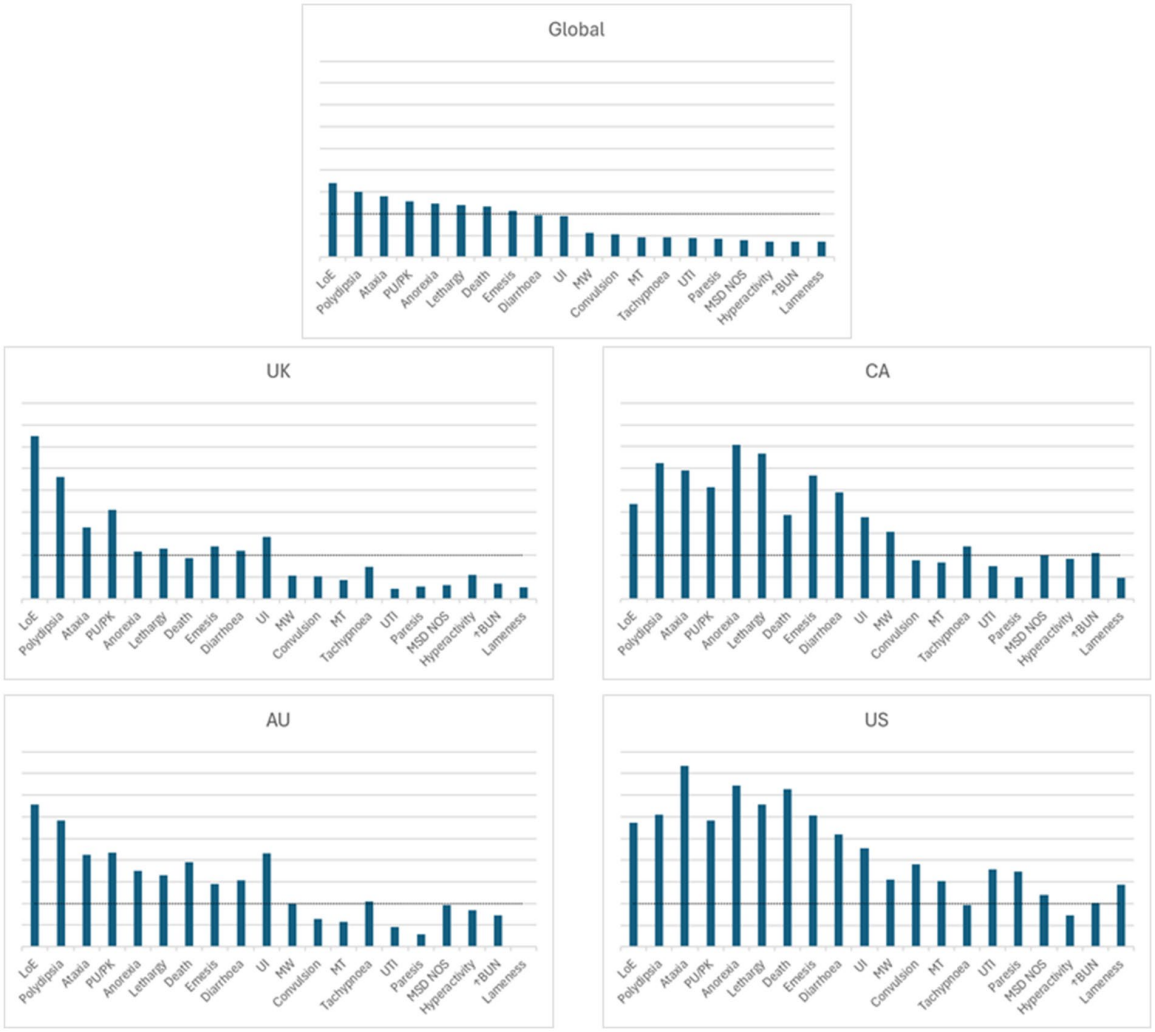
Visual representation of the frequency of adverse events for bedinvetmab global data (01 February 2021 to 30 June 2024) and for the top four countries by frequency of reporting (from launch until 30 June 2024). Each of the four countries was at a different stage of post-approval experience for bedinvetmab (United Kingdom (UK), 41 months; Canada (CA), 16 months; Australia (AU), 16 months; United States (US), 9 months). The Y axis refers to the number of events/10,000 treated dogs (doses). The X axis includes the top 20 reported events [Veterinary Dictionary for Drug Related Affairs (VeDDRA) terms] globally. For each country, the order of the VeDDRA terms is the same as for the global data. Terms with bars crossing the dotted line were reported at a frequency equal or greater than 1 event / 10,000 treated animals (doses) (i.e., considered to be rare) (22). The global pattern is the summation of different country patterns at different stages of commercialization. Note how the patterns of reporting differ from each other. LoE, Lack of Efficacy; MSD NOS, Musculoskeletal Disorder Not Otherwise Specified; MT, Muscle tremor; MW, Muscle Weakness; PU/PK, Polyuria/Pollakiuria; UI, Urinary Incontinence; UTI, Urinary Tract Infection; ↑BUN, Increased Blood Urea Nitrogen.

**Figure 5:**
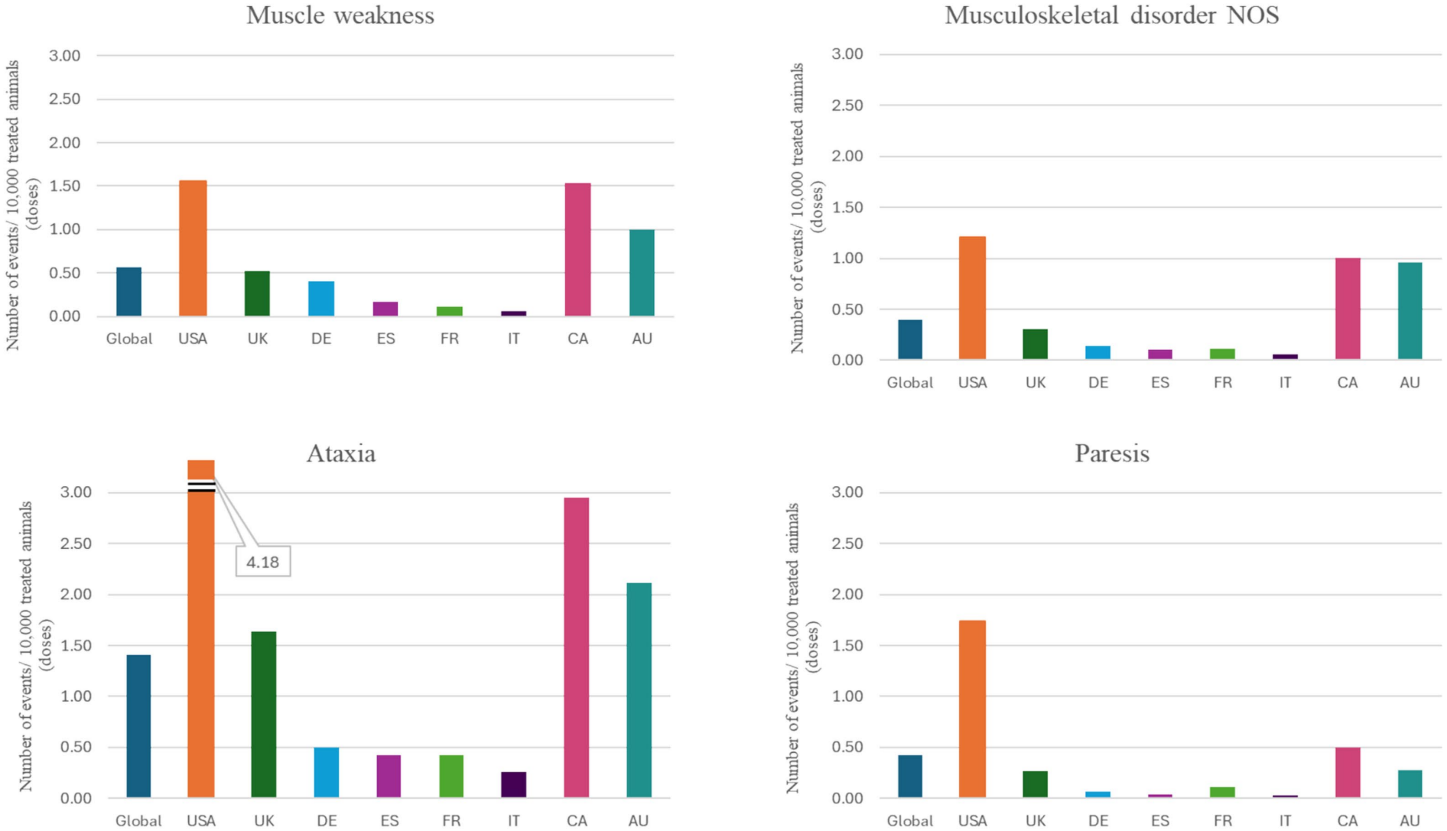
Individual VeDDRA (Veterinary Dictionary for Drug Related Affairs) terms related to musculoskeletal and neurological signs from 01 February 2021 to 30 June 2024, globally and for the top eight countries by market size for bedinvetmab. Note how the reporting frequency of these signs varies across different countries. CA, Canada; US, United States; UK, United Kingdom; DE, Germany; ES, Spain; FR, France; IT, Italy; CA, Canada; AU, Australia; NOS, Not otherwise specified.

The top eight countries by market size were US, UK, Germany, Spain, France, Italy, Canada, and Australia. From these, the top five countries by total number of adverse events were US, UK, Canada, Germany and Australia, and the top five by frequency of adverse events [number of events/10,000 treated animals (doses)] were Canada, US, UK, Australia and Germany (Figure 1). The type and frequency of adverse event reports varied across different countries (Table 2, Figures 4, 5).

**Table 2:** Heatmap of top 20 VeDDRA (Veterinary Dictionary for Drug Related Affairs) terms for bedinvetmab from 01 February 2021 to 30 June 2024, globally and for the top eight countries by market size.

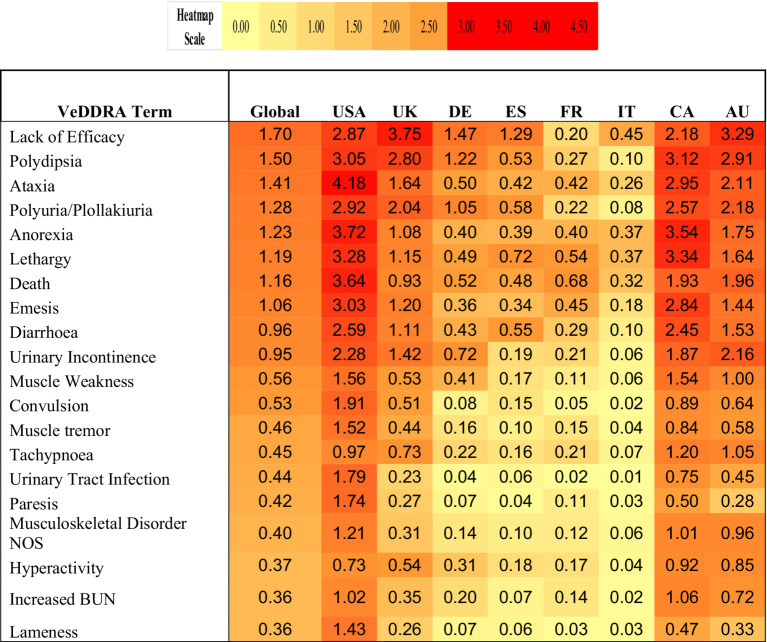

Reported numbers refer to the frequency of reported adverse events [number of events per 10,000 treated animals (doses)]. See Table 1 for description of categories of frequency of adverse drug reaction. CA, Canada; US, United States; UK, United Kingdom; AU, Australia; DE, Germany; ES, Spain; FR, France; IT, Italy; NOS, Not Otherwise Specified; BUN, Blood Urea Nitrogen.

## Discussion

This study found that the most common reported adverse events following the distribution of over 18 million doses of bedinvetmab are considered rare or very rare according to the definition by the CIOMS (22). It also showed variable frequencies across different countries. The frequency of reporting was not related to the size of the market. Most dogs for which adverse events were reported were considered older and in fair condition.

Results are presented based on reported adverse events independent of any assessment of causality. This means that if the adverse event was believed to be due to another VMP administered at the same time as bedinvetmab (e.g., an injection site reaction at the site of a vaccine administration rather than bedinvetmab administration) or to a pre-existing cause (e.g., a previously undiagnosed neoplasia), they are still included in the data herein. Causality is assigned by the MAH using the ABO_1_ON system (23, 24) which takes into account the associative connection, whether there is pharmacological and/or immunological explanation, presence of confirmatory information, previous knowledge of similar reports, exclusion of other causes and completeness / reliability of the reported data.

The mean age of dogs in this study was 11.4 years (compared to a mean of 4.7 years in the canine ZGPVDB). This is not surprising as the indication for use is the treatment of pain associated with OA which is currently predominantly treated in middle aged to older dogs. Similarly, dogs were predominantly assessed as being in ‘fair’ condition prior to treatment (compared to ‘good’ pre-treatment condition in the canine ZGPVDB). Compared to younger dogs, this older dog population likely has more comorbidities and progression of clinical signs associated with these comorbidities (25). Unfortunately, data do not exist across all countries to compare the bedinvetmab-treated population demographics with the population demographics of animals presenting an adverse event, but they are likely to be similar.

Lack of efficacy was reported in 1.70 out of 10,000 treated animals (doses). It includes any report related to lower-than expected efficacy or shorter duration of efficacy. Nearly half of the reports were related to decreased efficacy prior to the next dose (i.e., within 4 weeks after receiving a dose). As defined by the VICH, lack of efficacy is considered an adverse event, but not an adverse reaction. This adverse event was also reported in randomized clinical trials with bedinvetmab (26, 27). Lack of efficacy reports were based on observations/perceptions by either the veterinarians or the caregivers and could be related to several factors including a true lack of efficacy, shorter than expected duration of efficacy, presence of a condition that is not painful OA and will not respond to NGF sequestration, normal progression of OA, musculoskeletal injury due to rapid return to exercise or inability to correctly assess the level of OA pain. The latter is clearly a challenge in chronic pain management in animals. Although validated instruments for the assessment of OA-pain exist, clinical experience suggests that they are rarely used in practice and thus, pain assessment remains extremely subjective and influenced by numerous biases. Routine use of validated pain scales prior to and during treatment are strongly recommended for a clear understanding of the response to treatment in dogs with OA pain (28, 29). Recent recommendations include the use of home-based videos whereby dogs are filmed over time in the same area of the house performing the same activity, thus allowing for comparisons before and after treatment (28).

Reports of polyuria/pollakiuria, polydipsia, and urinary incontinence (PU/PD/UI) were rare but were disproportionately reported with bedinvetmab as compared to other canine products. These cases often described transient episodes of PU/PD/UI, with clinical presentation lasting a few days to weeks. They were of a reversible nature and did not appear to be associated with progressive renal impairment. Although the mechanistic pathophysiology for such signs currently remains unknown, the transient clinical characteristics of the reports suggests there is not an underlying pathological change in the urinary tract or endocrine system. Most reports of UI also included signs of PU and PD. It is possible that in at least some cases the former is a consequence of the latter in elderly dogs. Based on the information available, it was concluded that the evidence reviewed supports a potential causal association and this constitutes a new risk which occurs rarely following treatment with bedinvetmab. The product labeling was updated accordingly.

Reports of neurological signs were rare and included ataxia and convulsion/seizure, with ataxia reported most frequently. Ataxia is also frequently seen on the approved labels of commonly used NSAIDs (30–33). Ataxia is defined as disturbed coordination of movement and can be caused by dysfunction of the vestibular, cerebellar, or proprioceptive systems (34, 35). As such, without additional information on neurological findings it is difficult to further define the neuroanatomical source. Adverse event reports generally involved a description of the dog being ‘ataxic’ with no additional neurological findings and limited or no information on the neurologic status of the animal prior to bedinvetmab administration. Among dogs suffering from OA, a common sequela is muscle wasting and weakness which can be misinterpreted as ataxia, particularly when more than one limb is affected. Aged dogs with OA can suffer from numerous degenerative diseases that affect the peripheral and central nervous systems and that might not be recognized when lameness is severe. Bedinvetmab is a large molecule and is not expected to cross the blood brain barrier in individuals with a healthy blood brain barrier; thus, central effects of the drug are not expected to occur. Bedinvetmab has not been assessed in dogs with neurologic conditions because these patients were excluded from the clinical trials (26, 27). It is advised that pre-existing neurological conditions are identified and accounted for prior to treatment with bedinvetmab to not confound post treatment efficacy or suspect reaction assessment. From launch in Europe through March 2023, reports of ataxia were very rare [<1 event per 10,000 treated animals (doses)] at a time when more than 6 million doses had been distributed. In March 2023, the product was launched in Canada, and an increase was noted in reports of ataxia from April to June 2023. Following the US launch in September 2023, a notable increase in reports of ataxia occurred, even when considering increasing distribution. Between February 2021 and March 2023, public discussion on the internet regarding bedinvetmab and ataxia was relatively limited and focused primarily on efficacy and general safety profile, with only occasional mentions of neurological adverse events such as ataxia. From April 2023 to April 2024, discussion around reported adverse events increased significantly. This surge appeared to be influenced by the US FDA’s approval of bedinvetmab in May 2023, which expanded its availability and prompted broader discussions. A substantial rise was seen in articles, forum discussions, and social media posts addressing the association between bedinvetmab and ataxia, reflecting growing awareness and concern among pet caregivers and veterinary professionals. Notably, in April 2024, a Wall Street Journal article (36) highlighted adverse event reports, including cases of ataxia, leading to heightened scrutiny and more extensive online discourse. However, reports of ataxia with bedinvetmab remain rare [1–10 adverse events / 10,000 treated animals (doses)] in the US and globally and have demonstrated a notable increase from June to October 2024. It is possible that the increases in reports of ataxia in this period were influenced by notoriety bias (i.e., increased spontaneous reporting after a safety alert) (37, 38). Given the rare but consistent reporting, a causal relationship between bedinvetmab and ataxia could exist. There are ongoing efforts to evaluate this question. The product labeling was updated accordingly.

Gastrointestinal systemic signs including emesis and diarrhea are not infrequently reported in dogs of any age following any product administration, although anorexia is proportionally more commonly reported in older dogs. Indeed, in a recent study evaluating the prevalence of diseases in the overall dog population, including dogs of any age and body size, it was found that 14% of them might be affected by gastrointestinal signs (25). Although at a rare frequency and of ambiguous causality, anorexia continues to be one of the regularly reported events following bedinvetmab administration.

Although death and euthanasia have been reported after bedinvetmab administration, a causal relationship to the product has not been established. Unfortunately, most dogs with OA pain are only diagnosed and treated when they are older, and the disease is advanced (25). Thus, dogs being treated with bedinvetmab for OA pain are frequently affected by comorbidities and might be receiving concurrent medications. Nearly 80% of dogs experiencing an adverse event were either ≥10 years of age or of ‘unknown age’ and were considered to be in ‘fair’ condition prior to treatment with bedinvetmab. For dogs that may die or be euthanized after bedinvetmab treatment for any reason, including malignancy, caregivers often choose not to have necropsy performed and a conclusive cause of death can rarely be established.

In humans, OA is known to progress at different rates with three subgroups identified based on radiographs: stable, slow, and fast progression (39). There is similar, although limited, evidence that OA also progresses at different rates in dogs, with some showing faster radiographic progression influenced by several factors (40, 41); note that these dogs were not being treated with bedinvetmab. Progression of OA might be affected by use of anti-inflammatory drugs (42–46). In human patients, a condition called rapidly progressive OA (RPOA) was identified in the 1950s (47–50), and has been linked to various causes such as traumatic injury, septic arthritis, osteonecrosis, Charcot’s neuroarthropathy, and idiopathic rapidly destructive arthritis of the hip (51, 52). The condition gained renewed attention when high doses of tanezumab (a human anti-NGF mAb) administered in combination with NSAIDs long-term were linked to an increased risk of RPOA, and other destructive arthropathies in clinical trials in people (53–55). For these reasons, regulatory agencies (FDA; EMA) mandated that bedinvetmab labels include a statement about RPOA in humans while acknowledging that this condition has not been reported in dogs. Although individual musculoskeletal adverse events occur very rarely, the authors felt it was important to address the topic herein due to anecdotal reports of an unclassified arthropathy in a small number of dogs treated with bedinvetmab. When looking specifically at musculoskeletal adverse events or any reported event that included radiographs, a total of 2,404 cases were identified and carefully reviewed [1.33 events/10,000 treated animals (doses)]. Most of these cases (69%) described adverse events unrelated to orthopedic issues (e.g., the case file included radiographs to work up other conditions, not OA) and 6% had insufficient information for assessment. One quarter of cases (25%) were classified as either ‘lack of efficacy’ or ‘progression of OA’. Potential differential diagnoses for the latter cases include immune-mediated conditions, joint overuse, or rare conditions like humeral condylar fracture. Within musculoskeletal adverse event reports, none of the reviewed reports met the criteria for RPOA as described in humans. In rodent models of OA, both neutral and negative effects on the progression of OA have been reported, but RPOA itself has not been reported in an animal model (56–58). A recently published study by Merck Healthcare KGaA in a relatively acute rabbit cruciate transection / stifle instability model showed anti-NGF mAb-associated progressive arthropathy (56), but these findings do not align with the description of RPOA in humans and cannot be extrapolated to animals with naturally occurring OA. Despite the lack of evidence for a human RPOA-like syndrome in non-human animals, including dogs, Zoetis has partnered with specialists from different disciplines to comprehensively and continually investigate reports of unclassified arthropathies. The authors emphasize the importance of reporting all suspected cases for pharmacovigilance, ideally including pre- and post-treatment radiographs, patient activity levels, history of involved joint (trauma, infection, surgery, developmental disease, etc.), concurrent medications and body condition.

Bedinvetmab was first launched in the UK and the EU (including France, Germany and Spain) in February 2021, followed by Canada and Australia in March 2023, and the US in September 2023. Thus, these markets are at different stages of maturity. Pharmacovigilance data often fluctuate early in a product’s lifecycle as veterinarians and clients gain experience with its use. Over time, as the market matures, the data tend to stabilize. This explains some of the observed differences between markets, with the data reflecting input from both mature markets (UK, France, Germany and Spain, with 41 months of experience), maturing markets (Canada and Australia, with 16 months), and newer markets (US, with 9 months). Such differences could be related to the stage of the product on a market, cultural differences in reporting or the impact of social media. Temporal differences in reporting might also suffer from the ‘Weber effect’ which is characterized as adverse event reports for a new product peaking at the end of the second year after approval by a regulatory authority (59). However, the Weber effect is not yet particularly evident for bedinvetmab nor is it considered a consistent finding as it was not identified in the US FDA’s Adverse Event Reporting database when 62 human health drugs were reviewed (60).

Labels for VMPs can differ significantly between countries due to varying regulatory frameworks and local requirements. Each country or region has its own guidelines specifying what information must be included on the label or in the Summary of Product Characteristics. These guidelines cover not only key product details, such as composition, indications, and dosage, but also extend to safety information, and the inclusion of reported adverse events. Reporting rates for different VeDDRA terms in the top eight markets showed large variability (Table 2; Figures 4, 5). Two distinct patterns emerged. The first pattern, observed in the UK, Australia and Germany, showed lack of efficacy as the most frequently reported adverse event, followed by urinary tract-related signs (e.g., PU/PD/UI). Ataxia and gastrointestinal or systemic signs were reported less frequently in these countries. The second pattern, seen in the US, showed ataxia as the most frequently reported event, closely followed by gastrointestinal (e.g., emesis, diarrhea) and systemic signs (e.g., anorexia, lethargy). Canada exhibited a combination of these patterns, with higher rates of urinary, gastrointestinal, systemic, and ataxia-related signs, but lower lack of efficacy rates compared to the UK. Figure 5 shows the individual VeDDRA signs relevant to musculoskeletal and neurological signs and how their reporting frequency vary across different countries. The frequency of reports also varies considerably (Table 2). For example, reporting frequencies in Canada, US, UK, and Australia exceed the global average of 9.5 events per 10,000 treated animals (doses). In contrast, in Germany, the reporting frequency is below the global average, despite higher sales when compared to Canada and Australia (Figure 4). Another notable regional difference is that Canada, although being the seventh-largest market by size, it is first by frequency of adverse event reporting. Reasons for differences in reporting are not well understood but appear to be broadly consistent across different products. For example, reporting rates in Canada are consistently higher than other countries when looking at other products within the ZGPVDB and similar patterns have been anecdotally reported by other animal health pharmaceutical companies (AS, personal communications). It is difficult to speculate on the reasons for this. In general, high-income countries have higher reporting rates than low- and middle-income countries, (61) but all top eight countries herein are considered high-income. Undergraduate educational activities on the topic are different across countries and could influence reporting behaviors (62). It could also be that Canada’s higher reporting rates are related to education and awareness around the importance of pharmacovigilance reporting, as well and culture and regulatory engagement; however, given the limited evidence on the topic in veterinary medicine, this is only speculation at this time.

Being the first VMP of its class for the management of OA in dogs, bedinvetmab has been highly cited in social media including experiences with the product which cannot be scrutinized due to lack of valid medical information about such cases. The negative consequences of social media misinformation have been the subject of study in medical and social sciences and is unfortunately an issue for which modern society has yet to find practical solutions (63). It is possible that social media bias could have led to different reporting rates or VeDDRA term reporting in different countries. This and other biases should be considered when interpreting pharmacovigilance data. Misinformation or bias can lead to fearful pet caregivers declining treatment of their dogs despite favorable evidence of efficacy and safety of the product. This frustrates veterinary professionals and prevents dogs from being treated leading to suboptimal pain management and compromise of animal welfare (64). Clinical trials have demonstrated bedinvetmab’s efficacy and safety, showing statistically significant improvement in pain relief compared to placebo (26, 27). Bedinvetmab has been shown to significantly improve the QoL of dogs suffering from OA (65), and recently was demonstrated to have fewer clinical adverse events when compared with an NSAID over an 8-week treatment period (66). Recently published expert guidelines recommend both NSAIDs and anti-NFG mAbs as first line therapeutic options for the analgesic management of OA (28, 29, 67).

Pharmacovigilance is a multifaceted science that can reveal emerging safety or efficacy concerns, as well as potential product quality or manufacturing issues, for both new and existing products. The strengths of pharmacovigilance data include exposure of the VMP to a much larger and more diverse population when compared with clinical studies, and ability to track VMP over long periods offering a clearer view of long-term safety and efficacy and potentially identifying emerging patterns. However, pharmacovigilance data has limitations. They rely on spontaneous reporting and are inherently biased. Biases include reporting bias (e.g., underreporting with more serious adverse events being more likely to be reported, changes in reporting patterns over time, Weber effect) and notoriety bias (e.g., increase in the number of spontaneous reports after the publication of safety concerns via a communication channel; the increase in reports is not necessarily due to an increase in the reaction in question but rather to its identification and publication as being associated with the given drug) (37, 59, 68–70). Also, there are incomplete data sets (there is no one single source of all relevant information), poor data quality (frequently case histories provided are sparse and may not include all relevant information), and presence of confounding factors (e.g., comorbidities, underlying conditions, misdiagnosis or the use of concurrent medications that were not part of pivotal studies). In addition, pharmacovigilance reports include extra-label use and reports of lack of efficacy which could be partial or complete lack of efficacy. Finally, pharmacovigilance data include reports regardless of a confirmed causal relationship with the product.

Another challenge, as highlighted by the CIOMS, is the impossibility to estimate true incidence based on spontaneous reports (20). This occurs due to uncertainties about the true numerator, and difficulty in estimating the denominator (number of animals treated). Data across a range of different countries for disease incidence in the canine population at different ages or breeds is sparse to absent which means that calculating specific increased risk following the use of any VMP in a large population is not currently possible. The CIOMS recognizes this issue but still supports the use of estimation of frequency (Table 1) to help understand the safety profile of products post-launch. Despite the aforementioned limitations, when viewed in aggregate, the data from such reports still provide essential information on the safety and efficacy profile of the product. While this study was based on the data contained in the ZGPVDB, open sources of adverse event data are also provided by some regulatory authorities which should be useful for veterinarians in specific geographies. The FDA’s OpenFDA database^2^ contains all US origin reports. The EMA’s EVVet database^3^ contains what should be a very similar global dataset to that contained in the ZGPVDB (with the limitation that data on non-serious reports was not comprehensively collected in the EMA’s EVVet database for many VMPs prior to 28 January 2022).

Reporting of adverse events to pharmaceutical companies should be regarded as a collective responsibility of all stakeholders, including veterinary professionals, to help identify potential safety concerns of a VMP. In turn, pharmaceutical companies carefully analyze pharmacovigilance data, including the types of dogs that are being affected, while keeping an open mind about potential adverse events as well as meeting regulatory guidelines for individual case reporting, aggregate case reporting and notification of any identified changes in the benefit/risk of a VMP. Similarly, regulatory agencies have their role to play. These combined actions help to ensure the continued safety of a VMP and its availability to patients in need. The role of each stakeholder in this process cannot be underestimated.

Adequate patient selection and complete medical records including physical, orthopedic and neurological examination help to clarify whether clinical signs observed after treatment with a VMP could be treatment-related. As with any therapy, a risk–benefit analysis should guide clinical decision which should focus on analgesics and non-pharmacological therapies with proven efficacy with the ultimate goal of improving animals’ QoL.

In conclusion, this study found that the most reported adverse events in dogs following bedinvetmab are considered rare or very rare and highlights the importance of pharmacovigilance in identifying potential safety concerns of a VMP, particularly when such VMP is the first in its class for a specific condition. Reporting rates and patterns in general and for specific VeDDRA terms greatly vary within countries and are not related to market size. This study was not designed to identify risk factors and it remains unknown which patients are at risk of developing adverse events; yet, it should be noted that most dogs for which adverse events were reported were considered older and in fair condition and this population could be at increased risk of presenting with co-morbidities prior to or during treatment with bedinvetmab. This report provides insights to the most reported adverse events following bedinvetmab administration to dogs and highlights the value of veterinary professionals and caregivers in reporting pharmacovigilance cases to contribute to the understanding of the safety profile of a medicinal product.

## Data Availability

The data analyzed in this study is subject to the following licenses/restrictions: pharmacovigilance data are reported directly to regulatory authorities, in their respective jurisdictions, by the market authorization holder. The regulatory authority may make some of the case information publicly available. Requests to access these datasets should be directed to tony.simon@zoetis.com.

## Footnotes

^2^ https://open.fda.gov/

^3^ https://www.adrreports.eu/vet/

## References

[1] Enomoto M de Castro N Hash J Thomson A Nakanishi-Hester A Perry E. Prevalence of radiographic appendicular osteoarthritis and associated clinical signs in young dogs. Sci Rep. (2024) 14:2827. doi: 10.1038/s41598-024-52324-9 38310147 PMC10838335

[2] Wright A Amodie DM Cernicchiaro N. Identification of canine osteoarthritis using an owner-reported questionnaire and treatment monitoring using functional mobility tests. J Small Anim Pract. (2022) 63:609–18. doi: 10.1111/jsap.13500, PMID: 35385129 PMC9543207

[3] Robinson W Lepus C Wang Q. Low-grade inflammation as a key mediator of the pathogenesis of osteoarthritis. Nat Rev Rheumatol. (2016) 12:580–92. doi: 10.1038/nrrheum.2016.136, PMID: 27539668 PMC5500215

[4] Lascelles BD Gaynor JS Smith ES Roe SC Marcellin-Little DJ Davidson G. Amantadine in a multimodal analgesic regimen for alleviation of refractory osteoarthritis pain in dogs. J Vet Intern Med. (2008) 22:53–9. doi: 10.1111/j.1939-1676.2007.0014.x, PMID: 18289289

[5] Monteiro-Steagall BP Steagall PV Lascelles BD. Systematic review of nonsteroidal anti-inflammatory drug-induced adverse effects in dogs. J Vet Intern Med. (2013) 27:1011–9. doi: 10.1111/jvim.12127, PMID: 23782347

[6] Hunt JR Dean RS Davis GN Murrell JC. An analysis of the relative frequencies of reported adverse events associated with Nsaid Administration in Dogs and Cats in the United Kingdom. Vet J. (2015) 206:183–90. doi: 10.1016/j.tvjl.2015.07.025, PMID: 26361747

[7] Hefti FF Rosenthal A Walicke PA Wyatt S Vergara G Shelton DL. Novel class of pain drugs based on antagonism of NGF. Trends Pharmacol Sci. (2006) 27:85–91. doi: 10.1016/j.tips.2005.12.001, PMID: 16376998

[8] Abdiche YN Malashock DS Pons J. Probing the binding mechanism and affinity of Tanezumab, a recombinant humanized anti-Ngf monoclonal antibody, using a repertoire of biosensors. Protein Sci. (2008) 17:1326–35. doi: 10.1110/ps.035402.108, PMID: 18505735 PMC2492818

[9] Chang DS Hsu E Hottinger DG Cohen SP. Anti-nerve growth factor in pain management: current evidence. J Pain Res. (2016) 9:373–83. doi: 10.2147/jpr.S89061, PMID: 27354823 PMC4908933

[10] Enomoto M Mantyh PW Murrell J Innes JF Lascelles BDX. Anti-nerve growth factor monoclonal antibodies for the control of pain in dogs and cats. Vet Rec. (2019) 184:23. doi: 10.1136/vr.104590, PMID: 30368458 PMC6326241

[11] Isola M Ferrari V Miolo A Stabile F Bernardini D Carnier P. Nerve growth factor concentrations in the synovial fluid from healthy dogs and dogs with secondary osteoarthritis. Vet Comp Orthop Traumatol. (2011) 24:279–84. doi: 10.3415/VCOT-10-04-0051, PMID: 21674121

[12] Vincent TL Miller RE. Molecular pathogenesis of OA pain: past, present, and future. Osteoarthr Cartil. (2024) 32:398–405. doi: 10.1016/j.joca.2024.01.005, PMID: 38244717 PMC10984780

[13] VICH Steering Committee. VICH Guideline 24 Pharmacovigilance of veterinary medicinal products: management of Adverse Event Reports (Aers). VICH Steering Committee (2007). Available online at: https://vichsec.org/wp-content/uploads/2024/10/GL24-st7.pdf.

[14] World Health Organization. (2006). The safety of medicines in public health programmes: pharmacovigilance an essential tool. Available online at: https://www.who.int/publications/i/item/9241593911 (Accessed November 14, 2024).

[15] Signal Management Subcommittee AHI. (2024). A flexible signal management framework for animal health pharmacovigilance. Available online at: https://ahi.org/wp-content/uploads/Flexible-Signal-Management-Framework_2024_FINAL.pdf (Accessed November 14, 2024).

[16] Davies H Blackwell E Fins IS Noble PJM Pinchbeck G Pirmohamed M. Recording of suspected adverse drug reaction reporting in veterinary free-text clinical narratives. J Small Anim Pract. (2024) 65:361–7. doi: 10.1111/jsap.13721, PMID: 38441325

[17] Faasse K Porsius JT Faasse J Martin LR. Bad news: the influence of news coverage and Google searches on Gardasil adverse event reporting. Vaccine. (2017) 35:6872–8. doi: 10.1016/j.vaccine.2017.10.004, PMID: 29128382

[18] European Medicines Agency CVMP. Combined Veddra list of clinical terms for reporting suspected adverse events in animals and humans to veterinary medicinal products. Amsterdam, Netherlands: European Medicines Agency (2023).

[19] European Medicines Agency CVMP. Guidance notes on the use of Veddra terminology for reporting suspected adverse events in animals and humans. Amsterdam, The Netherlands: European Medicines Agency (2023).

[20] Council for International Organizations of medical sciences. (2024). Pharmacovigilance. Available online at: https://cioms.ch/pharmacovigilance/ (Accessed November 14, 2024).

[21] European Medicines Agency CfVMPC. Calculation of dose factor to be submitted to the union product database (Upd) - scientific guideline. Amsterdam, Netherlands: European Medicines Agency (2023).

[22] CIOMS Working Groups III and IV. Guidelines for preparing Core clinical-safety information on drugs. Geneva: Council for International Organizations of Medical Sciences (1999).

[23] European Medicines Agency. Recommendation on Harmonising the approach to causality assessment for adverse events to veterinary medicinal products. Amsterdam, Netherlands: European medicines agency (2013).

[24] Woodward KN. Pharmacovigilance for veterinary medicinal products In: Marrs T Woodward K, editors. Regulatory toxicology in the European Union. London: The Royal Society of Chemistry (2018). 243–354.

[25] Nam Y White M Karlsson EK Creevy KE Promislow DEL McClelland RL. Dog size and patterns of disease history across the canine age Spectrum: results from the dog aging project. PLoS One. (2024) 19:e0295840. doi: 10.1371/journal.pone.0295840, PMID: 38232117 PMC10793924

[26] Corral MJ Moyaert H Fernandes T Escalada M Kira STJ Walters RR. A prospective, randomized, blinded, placebo-controlled multisite clinical study of Bedinvetmab, a canine monoclonal antibody targeting nerve growth factor, in dogs with osteoarthritis. Vet Anaesth Analg. (2021) 48:943–55. doi: 10.1016/j.vaa.2021.08.001, PMID: 34565678

[27] Michels GM Honsberger NA Walters RR Kira STJ Cleaver DM. A prospective, randomized, double-blind, placebo-controlled multisite, parallel-group field study in dogs with osteoarthritis conducted in the United States of America evaluating Bedinvetmab, a canine anti-nerve growth factor monoclonal antibody. Vet Anaesth Analg. (2023) 50:446–58. doi: 10.1016/j.vaa.2023.06.003, PMID: 37541934

[28] Gruen ME Lascelles BDX Colleran E Gottlieb A Johnson J Lotsikas P. 2022 AAHA pain management guidelines for dogs and cats. J Am Anim Hosp Assoc. (2022) 58:55–76. doi: 10.5326/jaaha-ms-7292, PMID: 35195712

[29] Monteiro BP Lascelles BDX Murrell J Robertson S Steagall PVM Wright B. 2022 WSAVA guidelines for the recognition, assessment and treatment of pain. J Small Anim Pract. (2022) 64:177–254. doi: 10.1111/jsap.13566, PMID: 39985162

[30] American Academy of Veterinary Pharmacology and Therapeutics. Veterinary Clinical Drug Information Monographs - Meloxicam. (2004). Available from: https://cdn.ymaws.com/www.aavpt.org/resource/resmgr/imported/meloxicam.pdf (Accessed August 20, 2024).

[31] National Library of Medicine. Dailymed Deramaxx Post Approval Experience (Rev. 2010). (2010). Available from: https://dailymed.nlm.nih.gov/dailymed/drugInfo.cfm?setid=eced868b-c101-425a-8c5c-1a23ea43152f (Accessed August 20, 2024).

[32] National Library of Medicine. Dailymed Previcox Post Approval Experience (Rev. 2009). (2009). Available from: https://dailymed.nlm.nih.gov/dailymed/lookup.cfm?setid=e6d96ec6-04a4-4986-ac9d-82aa79b28bd4&version=2 (Accessed August 20, 2024).

[33] National Library of Medicine. Dailymed Rimadyl Post Approval Experience. Available from: https://dailymed.nlm.nih.gov/dailymed/drugInfo.cfm?setid=ae09baf6-1d34-44b0-b9c9-aa9766cdab14 (Accessed August 20, 2024).

[34] Thomas WB. Initial assessment of patients with neurologic dysfunction. Vet Clin N Am Small Anim Pract. (2000) 30:1–24. doi: 10.1016/S0195-5616(00)50001-1 10680207

[35] Garosi LS Lowrie M. The neurological examination In: Platt SR Olby NJ, editors. Bsava Manual of Canine and Feline Neurology. Quedgeley, UK: British Small Animal Veterinary Association (2013)

[36] Calfas J. What killed their pets? Owners blame meds, but vets aren’t sure. Wall Street J. (2024) 24:59.

[37] de Boissieu P Kanagaratnam L Abou Taam M Roux MP Dramé M Trenque T. Notoriety Bias in a database of spontaneous reports: the example of osteonecrosis of the jaw under bisphosphonate therapy in the French National Pharmacovigilance Database. Pharmacoepidemiol Drug Saf. (2014) 23:989–92. doi: 10.1002/pds.3622, PMID: 24737486

[38] Pariente A Gregoire F Fourrier-Reglat A Haramburu F Moore N. Impact of safety alerts on measures of disproportionality in spontaneous reporting databases the notoriety Bias. Drug Saf. (2007) 30:891–8. doi: 10.2165/00002018-200730100-00007, PMID: 17867726

[39] Kwoh CK Ran D Ashbeck EL Duryea J. Distinct trajectories of medial fixed joint space width loss over four years of follow-up among knees with and at risk for knee osteoarthritis. Arthritis Rheumatol. (2017) 69:2948.

[40] Enomoto M Baines EA Roe SC Marcellin-Little DJ Lascelles BDX. Defining the rate of, and factors influencing, radiographic progression of osteoarthritis of the canine hip joint Vet Rec. (2021) 189:e516. doi: 10.1002/vetr.516 34118160

[41] Innes JF Costello M Barr FJ Rudorf H Barr AR. Radiographic progression of osteoarthritis of the canine stifle joint: a prospective study. Vet Radiol Ultrasound. (2004) 96:143–8. doi: 10.1111/j.1740-8261.2004.04024.x 15072147

[42] Ding C. Do Nsaids affect the progression of osteoarthritis? Inflammation. (2002) 26:139–42. doi: 10.1023/A:1015504632021 12083420

[43] Doherty M. 'Chondroprotection' by non-steroidal anti-inflammatory drugs. Ann Rheum Dis. (1989) 48:619–21. doi: 10.1136/ard.48.8.619, PMID: 2675781 PMC1003833

[44] Newman NM Ling RSM. Acetabular bone destruction related to non-steroidal anti-inflammatory drugs. Lancet. (1985) 326:11–4. doi: 10.1016/S0140-6736(85)90058-3, PMID: 2861455

[45] Palmoski MJ Colyer RA Brandt KD. Marked suppression by salicylate of the augmented proteoglycan synthesis in osteoarthritic cartilage. Arthritis Rheum. (1980) 23:83–91. doi: 10.1002/art.1780230114, PMID: 7352948

[46] Palmoski MJ Brandt KD. In vivo effect of aspirin on canine osteoarthritic cartilage. Arthritis Rheum. (1983) 26:994–1001. doi: 10.1002/art.1780260808, PMID: 6882492

[47] Forestier F. Coxites Rhumatismales Subaigues Et Chroniques University of Paris (1957).

[48] Lequesne M. La Coxarthrose Destructrice Rapide. Rev Rhum Mal Osteoartic. (1970) 37:721–33. PMID: 5533642

[49] Postel M Kerboull M. Total prosthetic replacement in rapidly destructive arthrosis of the hip joint. Clin Orthop Relat Res. (1970) 72:138–44. doi: 10.1097/00003086-197009000-00016, PMID: 5459776

[50] Carrino JA McAlindon TE Schnitzer TJ Guermazi A Hochberg MC Conaghan PG. Characterization of adverse joint outcomes in patients with osteoarthritis treated with subcutaneous tanezumab. Osteoarthr Cartil. (2023) 31:1612–26. doi: 10.1016/j.joca.2023.08.010, PMID: 37652258

[51] Amstutz HC Le Duff MJ. The natural history of osteoarthritis: What happens to the other hip?. Clin Orthop Relat Res. (2016) 474:1802–9. doi: 10.1007/s11999-016-4888-y 27172820 PMC4925421

[52] Zazgyva A Gurzu S Gergely I Jung I Roman CO Pop TS. Clinico-radiological diagnosis and grading of rapidly progressive osteoarthritis of the hip. Medicine. (2017) 96:e6395. doi: 10.1097/md.0000000000006395 28328832 PMC5371469

[53] Hochberg MC. Serious joint-related adverse events in randomized controlled trials of anti-nerve growth factor monoclonal antibodies. Osteoarthr Cartil. (2015) 23:S18–21. doi: 10.1016/j.joca.2014.10.005, PMID: 25527216

[54] Hochberg MC Tive LA Abramson SB Vignon E Verburg KM West CR. When is osteonecrosis not osteonecrosis?: adjudication of reported serious adverse joint events in the Tanezumab clinical development program. Arthritis Rheumatol. (2016) 68:382–91. doi: 10.1002/art.39492, PMID: 26554876

[55] Food and Drug Administration. Joint meeting of arthritis advisory committee and drug safety and risk management advisory committee. Silver Spring, MD: Food and Drug Administration (2021).

[56] Menges S Michaelis M Kleinschmidt-Dorr K. Anti-Ngf treatment worsens subchondral bone and cartilage measures while improving symptoms in floor-housed rabbits with osteoarthritis. Front Physiol. (2023) 14:1201328. doi: 10.3389/fphys.2023.1201328, PMID: 37435308 PMC10331818

[57] Werts A Reece D Simon T Cole P. Re: re: laboratory safety evaluation of Bedinvetmab, a canine anti-nerve growth factor monoclonal antibody, in dogs. Vet J. (2024) 306:106175. doi: 10.1016/j.tvjl.2024.106175, PMID: 38885831

[58] Xu L Nwosu LN Burston JJ Millns PJ Sagar DR Mapp PI. The anti-Ngf antibody Mumab 911 both prevents and reverses pain behaviour and subchondral osteoclast numbers in a rat model of osteoarthritis pain. Osteoarthr Cartil. (2016) 24:1587–95. doi: 10.1016/j.joca.2016.05.015, PMID: 27208420 PMC5009895

[59] Weber JCP. Epidemiology of adverse reactions to nonsteroidal anti-inflammatory drugs In: Rainsford KD Velo GP, editors. *Advances in inflammation research*. 6. New York: Raven Press (1984). 1–7.

[60] Hoffman KB Dimbil M Erdman CB Tatonetti NP Overstreet BM. The Weber effect and the United States Food and Drug Administration's adverse event reporting system (Faers): analysis of sixty-two drugs approved from 2006 to 2010. Drug Saf. (2014) 37:283–94. doi: 10.1007/s40264-014-0150-2, PMID: 24643967 PMC3975089

[61] Garashi HY Steinke DT Schafheutle EI. A systematic review of pharmacovigilance Systems in Developing Countries Using the WHO pharmacovigilance indicators. Ther Innov Regul Sci. (2022) 56:717–43. doi: 10.1007/s43441-022-00415-y, PMID: 35657484 PMC9356965

[62] Hartman J Härmark L van Puijenbroek E. A global view of undergraduate education in pharmacovigilance. Eur J Clin Pharmacol. (2017) 73:891–9. doi: 10.1007/s00228-017-2237-z, PMID: 28314882

[63] Muhammed TS Mathew SK. The disaster of misinformation: a review of research in social media. Int J Data Sci Anal. (2022) 13:271–85. doi: 10.1007/s41060-022-00311-6, PMID: 35194559 PMC8853081

[64] Dobree L. The force of the internet. Vet Rec. (2024) 194:195. doi: 10.1002/vetr.4023

[65] Reid J Gildea E Davies V Thompson J Scott M. Measuring the effect of the anti-nerve growth factor antibodies Bedinvetmab and Frunevetmab on quality of life in dogs and cats with osteoarthritis using a validated health-related quality of life outcome measure: an observational real-world study. Front Vet Sci. (2024) 11:1395360. doi: 10.3389/fvets.2024.1395360, PMID: 39205806 PMC11349630

[66] Innes JF Lascelles BDX Bell D Tulloch R McVey A Northcott C. A randomised, parallel group clinical trial comparing bedinvetmab to meloxicam for the management of canine osteoarthritis. Frontiers Vet Sci. (2025) 12. doi: 10.3389/fvets.2025.1502218 PMC11974340 40196808

[67] Cachon TFO Innes JF Lascelles BDX Okumura M Sousa P Staffieri F. Coast development Group's international consensus guidelines for the treatment of canine osteoarthritis. Front Anim Sci. (2023) 10:1137888. doi: 10.3389/fvets.2023.1137888, PMID: 37601753 PMC10436090

[68] Hazell L Shakir SA. Under-reporting of adverse drug reactions. Drug Saf. (2006) 29:385–96. doi: 10.2165/00002018-200629050-00003, PMID: 16689555

[69] Moulis G Sommet A Durrieu G Bagheri H Lapeyre-Mestre M Montastruc JL. Trends of reporting of ‘serious’ vs.‘non-serious’ adverse drug reactions over time: a study in the French pharmacovigilance database. Br J Clin Pharmacol. (2012) 74:201–4. doi: 10.1111/j.1365-2125.2012.04185.x, PMID: 22257367 PMC3394146

[70] Matsuda S Aoki K Kawamata T Kimotsuki T Kobayashi T Kuriki H. Bias in spontaneous reporting of adverse drug reactions in Japan. PLoS One. (2015) 10:e0126413. doi: 10.1371/journal.pone.0126413, PMID: 25933226 PMC4416713

